# Impact of neoadjuvant and adjuvant radiotherapy on disease-specific survival in patients with stages II–IV rectal cancer

**DOI:** 10.18632/oncotarget.22460

**Published:** 2017-11-06

**Authors:** Yinying Wu, Haiyang Liu, Xianglin L. Du, Fan Wang, Jing Zhang, Xiaohai Cui, Enxiao Li, Jin Yang, Min Yi, Yunfeng Zhang

**Affiliations:** ^1^ Department of Medical Oncology, The First Affiliated Hospital of Xi’an Jiaotong University, Xi’an, Shaanxi, People’s Republic of China; ^2^ Department of Radiation Imaging, Shangluo Central Hospital, Shangluo, Shaanxi, People's Republic of China; ^3^ Department of Epidemiology, Human Genetics and Environmental Sciences, The University of Texas School of Public Health, Houston, TX, USA; ^4^ Second Department of Thoracic Surgery, The First Affiliated Hospital of Xi’an Jiaotong University, Xi’an, Shaanxi, People’s Republic of China; ^5^ Department of Breast Surgical Oncology, The University of Texas MD Anderson Cancer Center, Houston, TX, USA

**Keywords:** radiation therapy, neoadjuvant/adjuvant, disease specific survival, rectal cancer, tumor stage

## Abstract

**Objectives:**

The purposes of this study were to determine whether neoadjuvant or adjuvant radiotherapy affected disease-specific survival (DSS) in patients with rectal cancer and whether stratification by tumor stage affected the results.

**Results:**

55.5% patients had neoadjuvant-radiotherapy (NRT), and 18.3% patients had adjuvant- radiotherapy (ART). Multivariable models showed that treatment type was independently associated with DSS. Patients with stages III/IV tumors who received ART plus chemotherapy had significantly worse DSS than did those who received NRT plus chemotherapy (NCRT) (*P =* 0.03). Among patients with stage II tumors, those who received ART plus chemotherapy and those who received NCRT had similar DSS. Further stratification by risk group revealed that patients with stage IIIA tumors who received ART plus chemotherapy had significantly better DSS than did those who received NCRT (*P =* 0.04). The ART plus chemotherapy and NCRT groups had similar DSS in patients with stage IIA tumors. Among high-risk patients (T3N+/T4), the NCRT group had significantly better DSS than did the ART plus chemotherapy group. Patients who underwent surgery only had the worst DSS of all the treatment groups.

**Materials and Methods:**

From the Surveillance, Epidemiology, and End Results database, patients diagnosed with stages II–IV rectal cancer from 2004–2014 were identified. Clinicopathologic features, treatments, and DSS in different treatment groups were compared.

**Conclusions:**

NCRT or ART plus chemotherapy can reduce deaths from rectal cancer. Patients with stage IIIA tumors will benefit most from ART plus chemotherapy, whereas NCRT should be recommended to patients with stages II, IIIB, or higher tumors.

## INTRODUCTION

Colorectal cancer is the third most common cancer in the US and the third leading cause of cancer death [1]. Rectal cancer, which makes up nearly one-third of colorectal cancer cases [2], is often difficult to treat and carries a much higher risk of local recurrence.

Because of this high risk, radiotherapy is often added to the standard treatment for rectal cancer, surgery. By the early 1990s, on the basis of evidence from the Gastrointestinal Tumor Study Group and the National Surgical Adjuvant Breast and Bowel Project (NSABP), adjuvant chemotherapy and radiation therapy had become widely used in the US [3–5]. More recently, the availability of endorectal ultrasonography and new magnetic resonance imaging technologies has improved preoperative staging, making neoadjuvant chemo-radiotherapy (NCRT) the current standard of care for patients with locally advanced rectal cancer [6–8]. However, many patients with locally advanced rectal cancers still do not receive NCRT because their disease is understaged on preoperative imaging studies.

Adjuvant radiotherapy (ART) can reduce the risk of local recurrence by about 37% compared to surgery alone [9] and is therefore recommended for patients with T3, T4, or N+ rectal cancer [10–14]. However, it is not yet clear whether this translates into increased survival durations. The purpose of this study was to compare the impact of neoadjuvant radiotherapy (NRT) and ART on disease-specific survival (DSS) in patients with rectal cancer in a large population-based retrospective data set from the Surveillance, Epidemiology, and End Results (SEER) program. We also sought to determine whether tumor stage affected the relationship between NRT or ART and DSS.

## RESULTS

### Patient, tumor and treatment characteristics

A total of 28,589 patients were included in this study. Of these, 55.5% had NRT, 26.2% had surgery only, and 18.3% had ART. The median age at diagnosis was 61 years (mean, 61; range, 17–99). More than a third of patients (36.1%) had stage II tumors, 50.2% had stage III tumors, and 13.7% had stage IV tumors. The median tumor size was 4.2 cm. More than 70% of the patient population was non-Hispanic white, while 8.3% of the patients were black, 11.5% were Hispanic white, and 9.5% were Asian. More than two-thirds of the patients (69.2%) underwent partial proctectomy. About 81% of the included patients underwent chemotherapy as the first course of treatment.

The demographic and clinicopathologic characteristics of the patients in the 3 treatment groups (NRT, ART, and surgery only) are shown in Table 1. Younger patients (≤ 60 years old) received NRT significantly more often than did patients older than 60 (62.4% vs. 49.0%; *P* < 0.0001). More than 50% of patients in all but 3 subgroups underwent NRT: patients with stage IV tumors (36.5% had NRT), those with grade III or IV tumors (47.5% with grade III and 44.0% with grade IV had NRT), and those older than 60 (49.0% had NRT). Patients with stages II or III tumors were more likely to have been treated with NRT (more than 55%) than were patients with stage IV disease, who were more likely to have had surgery only (51.0%, *P* < 0.0001). NRT was more frequently used in patients who had total proctectomy (67.2%) than in those who had partial proctectomy (50.5%; *P* < 0.0001). Patients with stage T3 tumors were more likely to have had NRT than were patients with stages T1 and T2 tumors (58.5% vs. 38.5%; *P* < 0.0001). About two-thirds (67.0%) of patients who received chemotherapy as the first course of treatment also had NRT, whereas 84.2% of the patients who did not undergo chemotherapy as their first course of treatment had surgery only.

**Table 1:** Baseline demographic and clinicopathologic characteristics of the 28,589 study patients

	All patients, %	NRT, % (*N* = 15,870)	Surgery only, % (*N* = 7,494)	ART, % (*N* = 5,225)	*P* value
Age at diagnosis, years					0.0001
Mean (median)	61 (61)	59 (59)	66 (67)	61 (61)	
≤ 60	48.9	62.4	19.0	18.6	< 0.0001
> 60	51.1	49.0	33.1	17.9	
Sex					< 0.0001
Female	40.0	52.1	28.9	19.0	
Male	60.0	57.8	24.4	17.8	
Race					0.2
Non-Hispanic white	70.7	55.5	26.2	18.3	
Black	8.3	53.3	27.1	19.6	
Hispanic white	11.5	55.9	25.4	18.7	
Asian	9.5	55.6	27.3	17.1	
Insurance					< 0.0001^*^
Uninsured	3.0	66.3	19.1	14.6	
Any Medicaid	8.9	58.4	27.3	14.3	
Insured	60.1	60.1	23.9	16.0	
Unknown	28.0				
Tumor grade					< 0.0001^*^
I	5.9	55.9	25.5	18.6	
II	69.0	54.6	26.8	18.5	
III	14.8	48.0	30.4	21.6	
IV	1.5	44.0	33.5	22.5	
Unknown	8.7				
AJCC 6th edition TNM stage					< 0.0001
II	36.1	59.5	24.2	16.3	
III	50.2	57.8	20.9	21.3	
IV	13.7	36.5	51.0	12.5	
AJCC 6th edition T stage					< 0.0001^*^
T1/T2	11.2	38.5	33.0	28.5	
T3	77.0	58.5	24.6	16.9	
T4	10.6	54.2	28.0	17.8	
T0/NA/TX	1.2				
Lymph node status					< 0.0001
Negative	49.5	58.1	26.0	15.9	
Positive	60.5	54.2	26.0	19.8	
Surgery type					< 0.0001^*^
Partial proctectomy	69.2	50.5	29.4	20.1	
Total proctectomy	30.8	67.2	18.7	14.1	
Number of negative LN					< 0.0001^*^
< 12	46.3	57.8	24.9	17.3	
≥ 12	53.7	54.3	27.2	18.5	
Tumor size (cm)					0.0001
Mean (median)	4.8 (4.2)	4.7 (4)	5.2 (4.5)	4.6 (4.1)	
CEA					< 0.0001^*^
Normal	33.1	59.2	20.5	20.3	
Positive	30.0	57.4	27.6	15.0	
Borderline	0.4	54.8	26.9	18.3	
Unknown	36.5				0.01
Mucinous histology					
No	92.2	55.4	26.4	18.2	
Yes	7.8	57.1	23.5	19.4	
Chemotherapy					< 0.0001
No/Unknown	19.1	6.8	84.2	9.0	
Yes	80.9	67.0	12.5	20.5	

Abbreviations: NRT, neoadjuvant radiotherapy; ART, adjuvant radiotherapy; AJCC, American Joint Committee on Cancer; LN, lymph nodes; CEA, carcinoembryonic antigen. Percentages were calculated by row. ^*^P value calculated after exclusion of “unknown” category.

Table 2 shows the relationships between treatment modalities (radiotherapy, chemotherapy, and surgery), tumor stage, and risk classification. The majority of patients with stage II disease (57.9%) and stage III disease (56.7%) had NRT plus chemotherapy (NCRT), whereas significantly fewer (35.4%) patients with stage IV disease had NCRT (*P* < 0.0001). Surgery plus chemotherapy was much more common in patients with stage IV tumors (32.9%) than in those with stage III (8.4%) or stage II (3.8%) tumors. More than 18% of the total study cohort had received ART (16.3% of patients with stage II tumors, 21.3% of patients with stage III tumors, and 12.5% of patients with stage IV tumors). Very few patients with any stage of disease had NRT only (less than 2%) or ART only (less than 2.5%). On the basis of the results of the risk stratification by TNM stage in the Intergroup 0114 trial, we separated the study population into low-risk (stage IIIA: T1-2N1M0 or stage IIA: T3N0M0) and high-risk (T3N+ or T4 any N) groups [15]. Among the approximately 8% of patients with stage IIIA tumors, 40.3% underwent NRT and 29.3% underwent ART (Table 2). Among the 32.4% of patients with stage IIA tumors, 59.6% had NRT and 16.0% had ART. Among patients in high-risk group, 55.3% had NRT and 18.0% had ART.

**Table 2:** Relationships between disease stage, radiotherapy, and chemotherapy

	Stage II, *N* (%) (*N* = 10,319)	Stage III, *N* (%) (N = 14,353)	Stage IV, *N* (%) (*N* = 3,917)	*P* value
Treatment				< 0.0001
NCRT	5,976 (57.9)	8,132 (56.7)	1,388 (35.4)	
NRT only	164 (1.6)	168 (1.2)	42 (1.1)	
Surgery + chemo	396 (3.8)	1,211 (8.4)	1,290 (32.9)	
Surgery only	2,100 (20.4)	1,788 (12.5)	396 (10.1)	
ART + chemo	1,460 (14.1)	2,835 (19.8)	438 (11.2)	
ART only	223 (2.2)	219 (1.5)	50 (1.3)	
	Stage IIIA (*N* = 2,269)	Stage IIA (*N* = 9,269)	Others (*N* = 17,051)	
Treatment				< 0.0001
NCRT	895 (39.4)	5,375 (58.0)	9,226 (54.1)	
NRT only	19 (0.8)	148 (1.6)	207 (1.2)	
Surgery + chemo	294 (13.0)	338 (3.6)	2,265 (13.3)	
Surgery only	396 (17.5)	1,923 (20.7)	2,814 (16.5)	
ART + chemo	620 (27.3)	1,299 (14.0)	2,814 (16.5)	
ART only	45 (2.0)	186 (2.0)	261 (1.5)	

Abbreviations: NCRT, neoadjuvant chemo-radiotherapy; NRT, neoadjuvant radiotherapy; ART, adjuvant radiotherapy.

### Survival analysis

**Figure 1:**
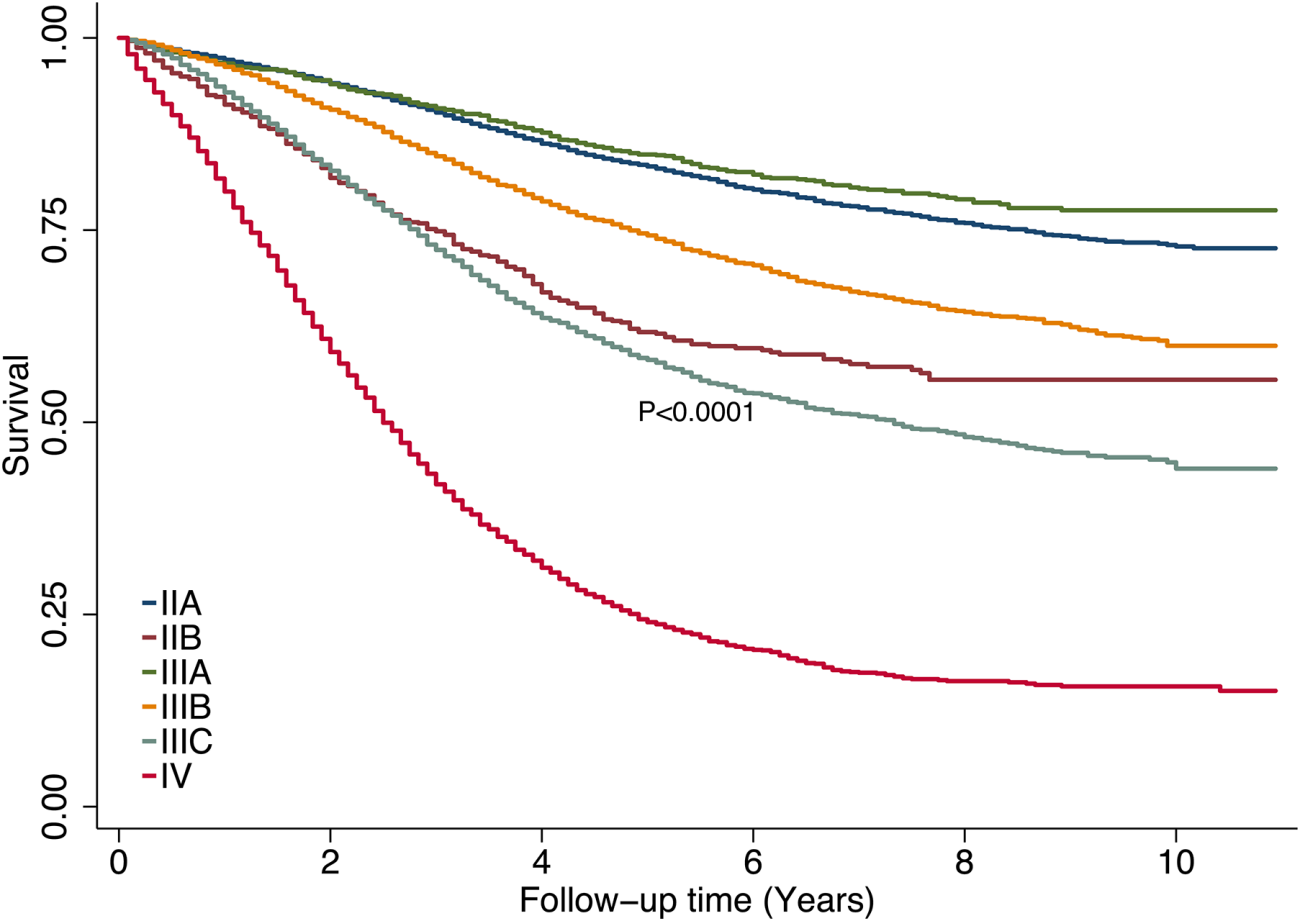
Disease-specific survival rates by tumor stage

The median follow-up time was 3.2 years (mean, 3.8; range, 0–10.9). Patients with low-risk (stages IIIA or IIA) tumors had better DSS than did patients with other stages (Figure 1).

We used Cox proportional hazards models to identify clinicopathologic factors related to DSS. We first performed this analysis for the entire patient cohort (Table 3). Older age (> 60 years), poorly differentiated tumor grade (III or IV), advanced tumor stage (III or IV), mucinous histology, carcinoembryonic antigen (CEA) positivity, and black race were associated with worse DSS. Moreover, the multivariable analyses confirmed that treatment types were independent factors associated with DSS: patients who received NCRT had the better DSS compared to patients who received other treatments. Some other factors, for example, sex and insurance status were not associated with DSS.

**Table 3:** Multivariable cox proportional hazards analysis of clinicopathologic factors associated with death from rectal cancer

Factor	HR	*P*	95% CI	
Treatment				
NCRT	Referent			
NRT only	1.3	0.005	1.1	1.6
Surgery + chemo	1.3	< 0.0001	1.2	1.3
Surgery only	2.0	< 0.0001	1.9	2.1
ART + chemo	1.1	0.002	1.0	1.2
ART only	1.8	< 0.0001	1.5	2.1
CEA				
Normal	Referent			
Positive	1.5	< 0.0001	1.45	1.6
Borderline	1.3	0.2	0.9	1.9
Unknown	1.3	< 0.0001	1.2	1.4
Age at diagnosis, years				
≤60	Referent			
>60	1.5	< 0.0001	1.4	1.6
Race				
Non-Hispanic white	Referent			
Black	1.3	< 0.0001	1.2	1.4
Hispanic white	1.1	0.06	1.0	1.2
Asian	0.9	0.02	0.8	0.98
Mucinous histology				
No	Referent			
Yes	1.5	< 0.0001	1.4	1.6
Surgery type				
Partial proctectomy	Referent			
Total proctectomy	1.3	< 0.0001	1.2	1.4
Tumor grade				
I	Referent			
II	1.1	0.1	1.0	1.2
III	1.7	< 0.0001	1.5	2.0
IV	2.3	< 0.0001	1.9	2.7
Unknown	1.1	0.4	0.9	1.2
AJCC 6th edition TNM stage				
II	Referent			
III	1.7	< 0.0001	1.6	1.8
IV	6.3	< 0.0001	5.9	6.7

Abbreviations: HR, hazard ratio; CI, confidence interval; NCRT, neoadjuvant chemo-radiotherapy; NRT, neoadjuvant radiotherapy; ART, adjuvant radiotherapy; CEA, carcinoembryonic antigen; AJCC, American Joint Committee on Cancer.

**Figure 2:**
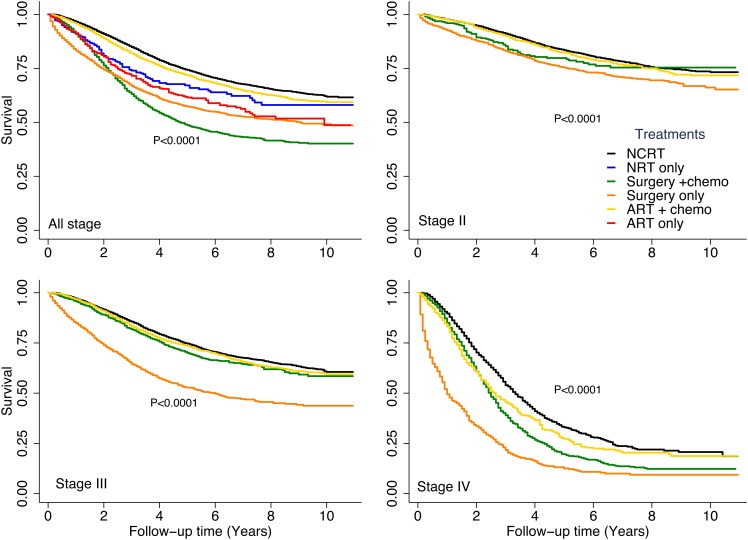
Disease-specific survival rates by treatment type stratified by tumor stage Subgroups with a sample size of less than 300 are not shown.

Figure 2 compares DSS durations among the treatment groups stratified by tumor stage. Patients in the surgery-only group had the shortest DSS of the stage-stratified treatment groups. Patients who received ART plus chemotherapy had worse DSS than did patients who received NCRT in whole cohort and in the stage-stratified stage III and stage IV cohorts.

Further analyses of the relationship between DSS and treatment modality in patients stratified by stage showed that when adjusted for disease stage, the patient’s age at diagnosis, tumor histologic grade, mucinous histology, and CEA status remained significant predictors of DSS (Table 4). In addition, in each stage-stratified cohort, patients who received surgery only (with or without chemotherapy) had significantly worse DSS than did patients who received NCRT. Among patients with stages III and IV disease, those who underwent ART plus chemotherapy had significantly worse DSS than did patients who underwent NCRT (*P* = 0.03). Among patients with stage II tumors, the risk of death from rectal cancer was similar in the ART plus chemotherapy and NCRT groups. Neither race nor type of surgery significantly predicted DSS in patients with stage IV disease.

**Table 4:** Multivariable cox proportional hazards analyses of clinicopathologic factors associated with death from rectal cancer, stratified by AJCC 6th edition TNM stage

Factor	Stage II (N = 10,319)				Stage III (N = 14,353)				Stage IV (N = 3,917)			
	HR	*P*	95% CI		HR	*P*	95% CI		HR	*P*	95% CI	
Treatment												
NCRT	Referent											
NRT only		–				–				-		
Surgery + chemo	1.3	0.03	1.02	1.7	1.2	0.001	1.1	1.4	1.3	< 0.0001	1.2	1.5
Surgery only	1.5	< 0.0001	1.3	1.7	2.1	< 0.0001	1.9	2.3	2.6	< 0.0001	2.3	2.9
ART + chemo	1.1	0.1	1.0	1.3	1.1	0.03	1.01	1.2	1.2	0.03	1.0	1.3
ART only		–				–				-		
CEA												
Normal	Referent											
Positive	1.5	< 0.0001	1.4	1.8	1.6	< 0.0001	1.4	1.7	1.5	< 0.0001	1.3	1.7
Borderline	1.5	0.3	0.7	3.1	1.2	0.5	0.7	1.9	1.6	0.3	0.7	3.9
Unknown	1.3	< 0.0001	1.2	1.5	1.3	< 0.0001	1.2	1.4	1.3	< 0.0001	1.1	1.5
Age at diagnosis, years												
≤60												
>60	1.6	< 0.0001	1.5	1.8	1.5	< 0.0001	1.4	1.6	1.3	< 0.0001	1.2	1.4
Race												
Non-Hispanic white	Referent											
Black	1.4	< 0.0001	1.2	1.6	1.3	< 0.0001	1.2	1.5		NS		
Hispanic white	1.1	0.4	0.9	1.3	1.2	0.03	1.1	1.3		NS		
Asian	0.8	0.08	0.7	1.0	0.9	0.1	0.8	1.0		NS		
Mucinous histology												
No	Referent											
Yes	1.4	< 0.0001	1.2	1.7	1.5	< 0.0001	1.4	1.7	1.4	< 0.0001	1.3	1.6
Surgery type												
Partial proctectomy	Referent											
Total proctectomy	1.2	< 0.0001	1.1	1.4	1.4	< 0.0001	1.3	1.5		NS		
Tumor grade												
I	Referent											
II	1.0	0.7	0.9	1.3	1.2	0.08	1.0	1.4	1.0	0.9	0.8	1.3
III	1.7	< 0.0001	1.4	2.2	1.8	< 0.0001	1.5	2.1	1.7	< 0.0001	1.3	2.1
IV	1.5	0.1	0.9	2.4	2.5	< 0.0001	1.9	3.2	2.3	< 0.0001	1.7	3.2
Unknown	1.1	0.3	0.9	1.5	1.1	0.5	0.9	1.3	1.0	0.8	0.8	1.3

Abbreviations: HR, hazard ratio; CI, confidence interval; NCRT, neoadjuvant chemo-radiotherapy; NRT, neoadjuvant radiotherapy; ART, adjuvant radiotherapy; CEA, carcinoembryonic antigen; -, not reported due to small sample size (*N* < 250); NS, not significant.

**Figure 3:**
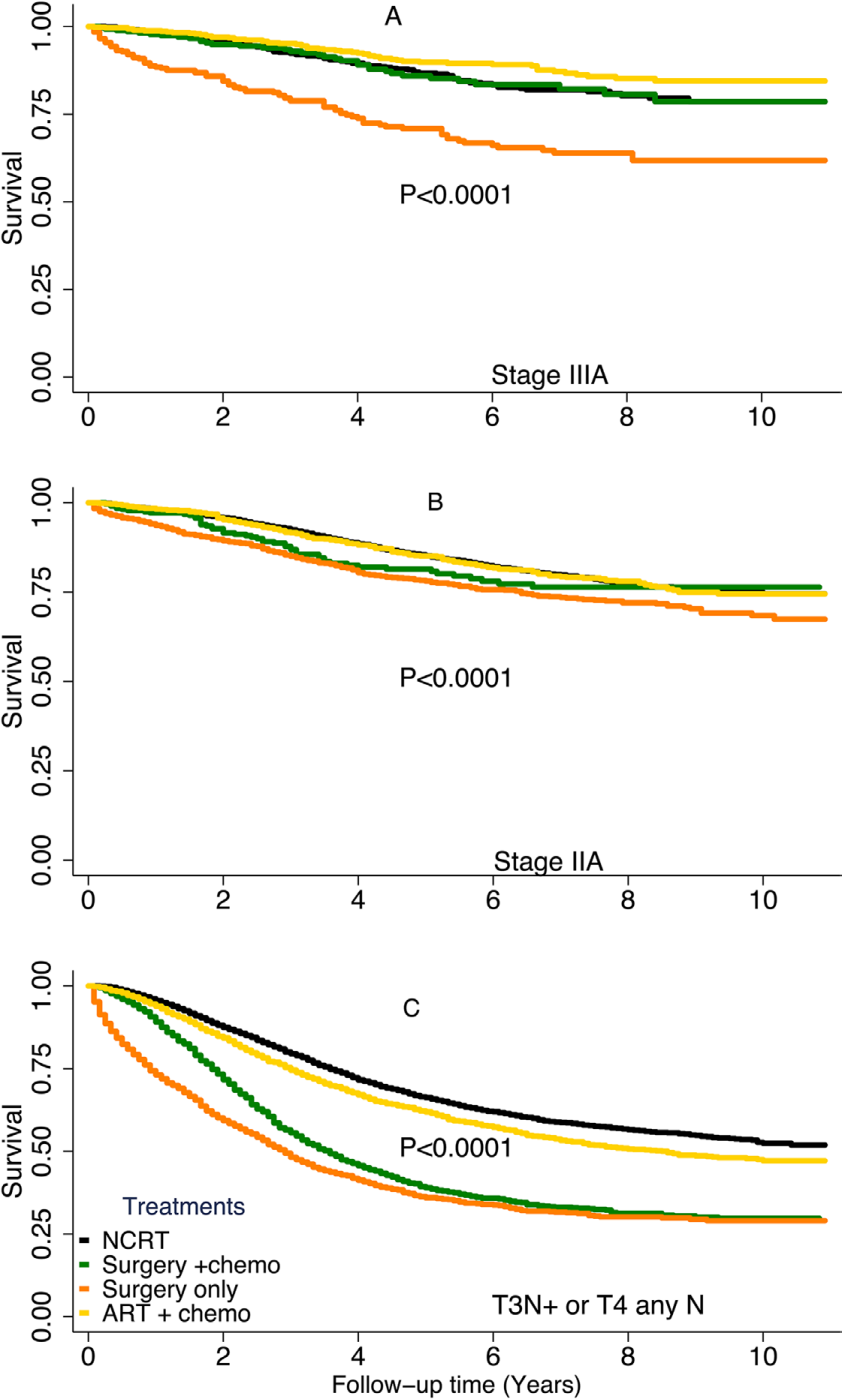
Disease-specific survival rates by treatment type stratified by risk group (**A**), stage IIIA, (**B**), stage IIA, (**C**), T3N+ or T4 any N. Subgroups with a sample size of less than 300 are not shown.

Among patients with stage IIIA tumors (Figure 3A), those who received ART plus chemotherapy had significantly better DSS than did those who received NCRT (hazard ratio [HR], 0.7; *P* = 0.04). Patients with stage IIIA tumors who underwent surgery only had the worst DSS of all the treatment groups (HR, 1.9; *P* < 0.00001). Patients with stage IIIA tumors who received surgery plus chemotherapy had DSS durations similar to those of patients who received NCRT. Among patients with stage IIA tumors (Figure 3B), those who received ART plus chemotherapy, those who received surgery plus chemotherapy, and those who received NCRT had similar DSS rates. Patients with stage IIA tumors who received surgery only had the worst DSS of the treatment groups (HR, 1.4; *P* < 0.00001).

In patients with high-risk tumors (Figure 3C), the NCRT group had the best DSS of the treatment groups. Patients who received surgery with or without chemotherapy had the worst DSS of the patients in this risk group. When we broke down the high-risk group to stage IIB (*N* = 1,036), stage IIIB (*N* = 7,860), stage IIIC (*N* = 4,143), we found the similar trends in those groups. Multivariable Cox proportional hazards analyses stratified by TNM risk groups demonstrated that the clinicopathologic factors associated with risk of death from rectal cancer were similar in all 3 groups (Table 5). The patient’s age at diagnosis, tumor histologic grade, mucinous histology, and CEA status remained significant predictors of DSS.

**Table 5:** Multivariable cox proportional hazards analyses of clinicopathologic factors associated with death from rectal cancer, stratified by TNM Risk Groups

Factor	Stage IIIA (*N* = 2,269)				Stage IIA (*N*=9,269)				Others (*N* = 17,051)			
	HR	*P*	95% CI		HR	P	95% CI		HR	*P*	95% CI	
Treatment												
NCRT	Referent											
NRT only		–				–				-		
Surgery + chemo	1.0	0.9	0.7	1.5	1.3	0.06	1.0	1.7	2.1	< 0.0001	2.0	2.3
Surgery only	1.9	< 0.0001	1.4	2.6	1.4	< 0.0001	1.2	1.6	2.7	< 0.0001	2.5	2.9
ART + chemo	0.7	0.04	0.5	0.98	1.0	0.6	0.9	1.2	1.2	< 0.0001	1.1	1.3
ART only		-				-				-		
CEA												
Normal	Referent											
Positive	1.6	0.005	1.2	2.3	1.5	< 0.0001	1.3	1.7	1.8	< 0.0001	1.7	1.9
Borderline	0.9	0.9	0.1	6.5	1.4	0.4	0.6	3.2	1.1	0.6	0.7	1.8
Unknown	1.4	0.005	1.1	1.9	1.3	< 0.0001	1.2	1.5	1.4	< 0.0001	1.3	1.5
Age at diagnosis, years												
≤60												
>60	2.1	< 0.0001	1.6	2.7	1.7	< 0.0001	1.5	1.9	1.3	< 0.0001	1.2	1.4
Race												
Non-Hispanic white	Referent											
Black	1.0	0.8	0.7	1.6	1.4	< 0.0001	1.2	1.7	1.2	< 0.0001	1.1	1.3
Hispanic white	1.5	0.02	1.1	2.1	1.1	0.2	0.9	1.3	1.0	0.5	0.9	1.1
Asian	0.9	0.6	0.6	1.3	0.8	0.04	0.7	0.99	0.9	0.04	0.8	1.0
Mucinous histology												
No	Referent											
Yes	1.1	0.7	0.7	1.8	1.4	< 0.0001	1.2	1.7	1.4	< 0.0001	1.3	1.5
Surgery type												
Partial proctectomy	Referent											
Total proctectomy	1.6	< 0.0001	1.2	2.0	1.2	0.002	1.1	1.3	1.2	< 0.0001	1.1	1.3
Tumor grade												
I	Referent											
II	0.9	0.7	0.6	1.4	1.0	0.8	0.8	1.3	1.1	0.1	1.0	1.3
III	1.2	0.4	0.7	2.1	1.7	< 0.0001	1.4	2.2	1.7	< 0.0001	1.5	2.0
IV	3.3	0.002	1.6	7.1	1.3	0.3	0.7	2.4	2.2	< 0.0001	1.8	2.7
Unknown	0.7	0.3	0.4	1.4	1.1	0.6	0.8	1.4	1.2	0.07	1.0	1.4

Abbreviations: HR, hazard ratio; CI, confidence interval; NCRT, neoadjuvant chemo-radiotherapy; NRT, neoadjuvant radiotherapy; ART, adjuvant radiotherapy; CEA, carcinoembryonic antigen; -, not reported due to small sample size (*N* < 250).

**Figure 4:**
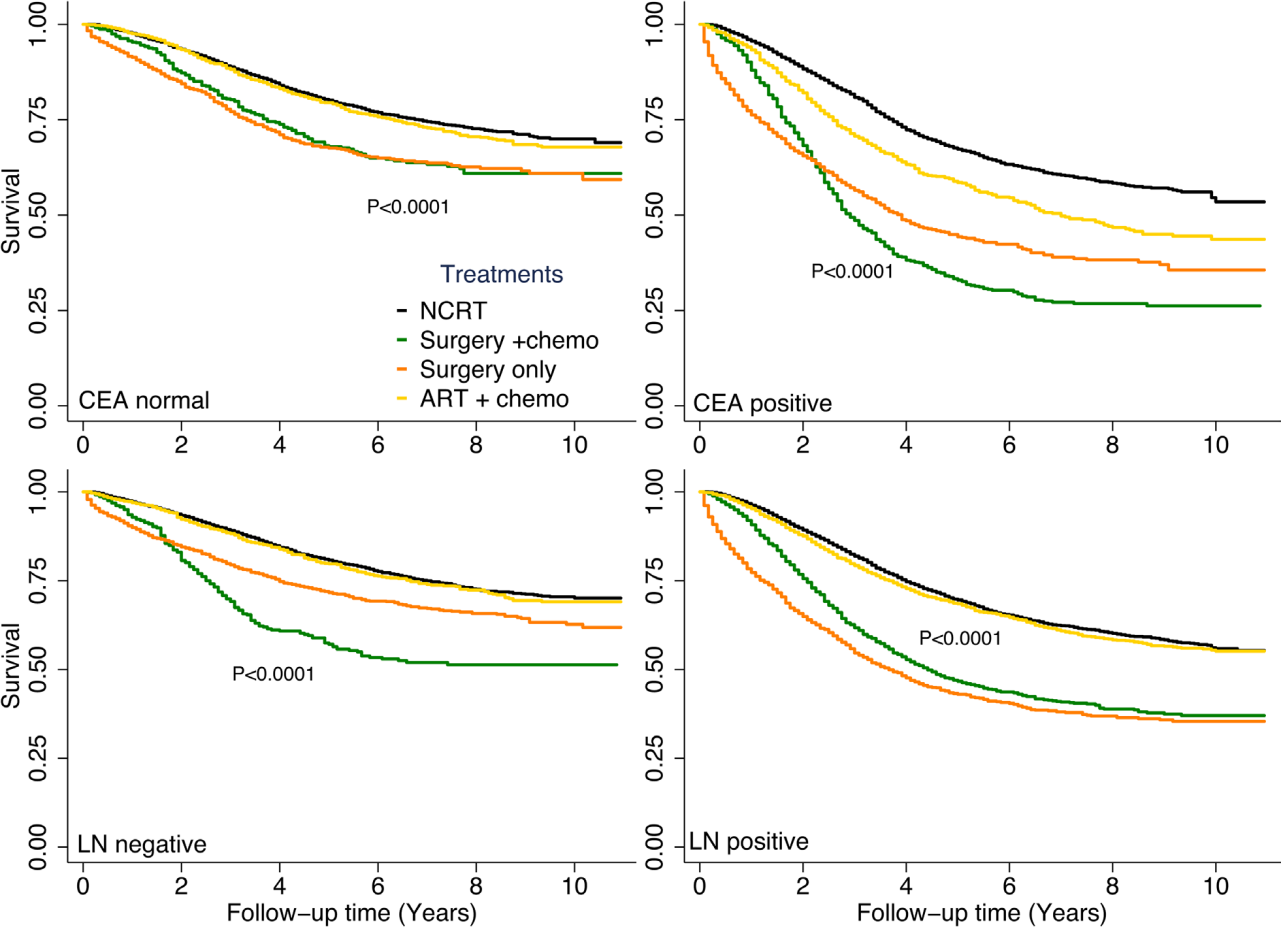
Disease-specific survival rates by treatment type stratified by carcinoembryonic antigen (CEA) level or lymph node (LN) status Subgroups with a sample size of less than 300 are not shown.

Figure 4 illustrates DSS rates among different treatment groups stratified by CEA levels or lymph node status. Among patients with normal CEA levels, patients who received ART plus chemotherapy and those who received NCRT had similar DSS (HR, 1.0; *P* = 0.3). In both the positive and negative lymph node groups, patients who received ART plus chemotherapy and patients who received NCRT had similar DSS rates. In patients with positive CEA tests, those who received ART plus chemotherapy had worse DSS than did those who received NCRT (HR, 1.1; *P* < 0.0001).

Table 6 shows 5-year survival rates by tumor risk groups and treatment groups. Among patients with stages IIA or IIIA tumors, those who received NCRT had 5-year overall survival (OS) rates of 79.0% and 81.0%, respectively. Patients with stages IIA or IIIA tumors who received ART plus chemotherapy had 5-year OS rates of 78.4% and 86.6%, respectively (*P* < 0.05). Patients who underwent surgery only had the worst 5-year OS rates, 60.8% for stage IIA and 56.5% for stage IIIA. Patients with stages IIA or IIIA tumors who received NCRT had 5-year DSS rates of 85.2% and 86.6%, respectively. Those who underwent ART plus chemotherapy had 5-year DSS rates of 85.1% for stage IIA and 89.8% for stage IIIA, and those who underwent surgery only had the worst 5-year DSS rates, 78.1% and 70.9%, respectively.

**Table 6:** Five-year survival rates by tumor risk group and treatment

		IIA	IIIA	Others
OS, % (95% CI)	NCRT	79.0 (77.6–80.3)	81.0 (77.7–84.0)	61.9 (60.6–63.1)
	Surgery + chemo	74.1 (68.2–79.1)	81.8 (75.8–86.4)	35.0 (32.6–37.4)
	Surgery only	60.8 (58.3–63.2)	56.5 (50.6–61.9)	26.3 (24.2–28.4)
	ART + chemo	78.4 (75.7–80.1)	86.6 (83.2–89.3)	57.1 (55.0–59.1)
DSS, % (95% CI)	NCRT	85.2 (83.9–86.3)	86.6 (83.6–89.1)	66.3 (65.0–67.5)
	Surgery + chemo	81.4 (75.9–85.8)	85.9 (80.2–90.9)	39.0 (36.5–41.5)
	Surgery only	78.1 (75.7–80.2)	70.9 (65.0–75.9)	36.0 (33.5–38.5)
	ART + chemo	85.1 (82.7–87.2)	89.8 (86.7–92.3)	61.9 (59.8–63.9)

Abbreviations: NCRT, neoadjuvant chemo-radiotherapy; NRT, neoadjuvant radiotherapy, ART, adjuvant radiotherapy; OS, overall survival; CI, confidence interval; DSS, disease-free survival.

For patients with stage IIIA tumors, treatment with ART plus chemotherapy improved 5-year DSS by 3.2% and 5-year OS by 5.6% over treatment with NCRT. Treatment with ART plus chemotherapy also improved 5-year DSS by 18.9% and 5-year OS by 30% over surgery only. Among patients with stage IIA tumors, treatment with ART plus chemotherapy and treatment with NCRT yielded similar 5-year DSS rates, and both treatment modalities improved 5-year DSS by 7% over surgery only. However, patients in the high-risk group who received NCRT had higher 5-year DSS rates than did those who received ART plus chemotherapy.

## DISCUSSION

The current study represents one of the most comprehensive population-based analyses of the impact of NRT and ART on the risk of death from rectal cancer by tumor stage and other potential confounders. Our study found that ART and NCRT can reduce death from rectal cancer. This result is consistent with the results of other studies [16–18], including the Swedish Rectal Cancer Trial, which was the first to find that NRT significantly decreased local recurrence rates and improved OS rates [18]. The results of several meta-analyses also support the use of NRT or ART with surgery to improve local disease control and survival rates [9, 19, 20].

Currently, NCRT is the standard of care for patients with locally advanced rectal cancer [4, 5, 10]. However, because of limitations in the imaging modalities used for preoperative staging, some patients are understaged. For these patients, ART is particularly important. Our study found that more than 18% of patients had received ART (16.3% for stage II, 21.2% for stage III, 12.5% for stage IV). Studies have demonstrated that the use of ART plus chemotherapy improved outcomes for patients with locally advanced rectal cancer [5]. However, the German CAO/ARO/AIO 94 trial found that NCRT improves local recurrence more than ART plus chemotherapy, but no effect on overall survival [6].

In this study, patients with stages IIA or IIIA tumors were grouped into a low-risk group, on the basis of the risk determinations made by the Intergroup 0114 trial [15]. Our findings confirmed that patients with stages IIA or IIIA tumors formed a low-risk group that had better DSS than other stage groups [15, 21–23]. We also found that a subset of these low-risk patients benefitted from ART plus chemotherapy; patients with stage IIIA tumors who received ART plus chemotherapy had better DSS than did those who received NCRT. These findings differ from those of Gunderson *et al.* [23], who found that patients receiving surgery plus chemotherapy had similar 5-year OS rates as those who received surgery plus chemoradiation (78% vs. 83%, respectively). However, the numbers of patients in each stage subset in that study were small. Our SEER-based study had a much larger sample: 2,269 patients had stage IIIA tumors, 9,269 patients had stage IIA tumors, and 17,051 patients had tumors of other stages. And in our study, we also adjusted the impact of treatments on survival with other biological factors: age, race, CEA, tumor grade and histology, which helped better estimate the progression. In a study of NRT in patients with locally advanced rectal cancer, Sauer *et al.* [24] found that 18% of the patients with clinical stage II/III disease diagnosed using endorectal ultrasonography who did not undergo NRT had stage I disease on surgical specimen, suggesting that ultrasonography staging may result in overtreatment in some patients. Therefore, for patients with low risk (stage IIIA disease), ART plus chemotherapy may be a better treatment compared to NCRT.

We found that patients with stage IIA tumors who received ART plus chemotherapy and those who received surgery plus chemotherapy had similar DSS durations as patients who received NCRT. We also found that patients with high-risk tumors who received NCRT had significantly longer DSS than did those who received ART plus chemotherapy. Our results are consistent with those published by Gunderson *et al.* [23]. NCRT is considered the preferred approach because of its lower morbidity rate. Sauer and colleagues [6] found fewer/lower toxicities in patients who received NCRT (27 vs 40%), and the 5-year and 10-year local recurrence (LR) rates were similar. Similarly, the NSABP R-03 trial found significant improvement in 5-year disease-free survival rates for patients receiving NCRT compared to patients receiving adjuvant chemoradiotherapy [7]. On the basis of those studies, NCRT is considered the preferred approach for patients with stage IIA tumors because it carries a lower incidence of morbidities.

This study has some limitations. First, its retrospective population-based design carries an unavoidable risk of selection bias. Second, data for some factors, including tumor grade, CEA level, and surgery type were not available for all patients. This may have influenced the comparison of the subgroups with the total population. Third, 2 main limitations affect analyses using SEER radiotherapy and chemotherapy data: (1) the completeness of the variables, some data are missing for radiation therapy and chemotherapy, and (2) biases associated with unmeasured reasons for receiving or not receiving radiotherapy or chemotherapy. Fourth, some information was not included in the SEER database—for example, chemotherapy regimen and cycles, protocol of radiotherapy, rates of recurrence and toxicities, which could affect survival calculations. However, our study comprehensively analyzed the impact of radiotherapy on death from rectal cancer by tumor stage. The potential relationships of other prognostic factors, such as ethnicity, histology, and CEA levels are complex and could be significant. Our findings may help to understand the biological behavior of rectal tumors and to identify patients at high risk of rectal cancer death in clinical practice.

In conclusion, neoadjuvant or adjuvant radiotherapy plus chemotherapy can reduce deaths from rectal cancer. Patients with stage IIIA tumors are appropriate candidates for ART plus chemotherapy, and NCRT should continue to be recommended to patients with stage II, stage IIIB, or more advanced tumors. To balances clinical benefits and treatment-related toxicities, randomized prospective trials are needed to develop an individualized treatment for advanced rectal cancer.

## MATERIALS AND METHODS

### Patient selection and data collection

The data were obtained from all 18 US cancer registries included in the SEER database (National Cancer Institute) by using the SEER*Stat software program (version 8.3.4; http://seer.cancer.gov/seerstat [accessed April, 26, 2017]) under a data user agreement. Patient records were anonymized and de-identified prior to analysis. Because the data were de-identified and obtained from a third party, no ethics committee review approval was required. The SEER database was searched to identify patients whose primary tumor sites were coded as C20.9 (rectum) and whose cancers were diagnosed from 2004 to 2014. Patients whose first primary malignancy was stage II-IV rectal cancer according to the 6^th^ edition of the American Joint Committee on Cancer staging guidelines were included. Patients who had not undergone surgery for resection of the primary tumor were excluded. Patients who had undergone radiation therapy before or after surgery and those who did not undergo radiotherapy were included. From the SEER database, we extracted data on patient demographics, primary tumor site, tumor morphology, cancer stage at diagnosis, first course of treatment, follow-up vital status and other clinical characteristics.

### Statistical analysis

Our primary interest was whether NRT or ART reduced deaths from rectal cancer after adjustment for clinicopathologic factors. The primary endpoint of this study was DSS, which was defined as the number of years from the date of rectal cancer diagnosis to the date of cancer-related death, the date on which the patient was last known to be alive, or November 30, 2014, whichever came first. DSS curves were calculated using the Kaplan-Meier method. Patients who died during follow-up or survived beyond November 30, 2014, were censored.

Patients were divided into 3 groups: NRT, surgery only, and ART. A chi-square test was used to assess differences in categorical variables, and a Kruskal-Wallis equality-of-populations rank test was used to assess differences in continuous variables. Multivariable Cox proportional hazards models were used to determine the influence of patient, tumor, and treatment characteristics of known or potential prognostic value (age at diagnosis, sex, year of diagnosis, ethnicity, cancer stage, tumor grade, surgery type, CEA level, and lymph node status) on DSS. The Stata/SE software program (version 12; StataCorp, College Station, TX, USA) was used for statistical analyses. All tests were 2-tailed, and statistical significance was set at *P* < 0.05.

## Abbreviations

DSS: disease specific survival
NRT: neoadjuvant radiotherapy
ART: adjuvant radiation therapy
NCRT: neoadjuvant chemo-radiotherapy
CEA: Carcinoembryonic Antigen
HR: Hazard ratio
OS: overall survival
SEER: Surveillance, Epidemiology, and End Results
